# Adding Concurrent Chemotherapy Significantly Improves the Survival of Stage II-IVb Nasopharyngeal Carcinoma Patients Treated With Concurrent Anti-EGFR Agents

**DOI:** 10.3389/fonc.2021.814881

**Published:** 2021-12-17

**Authors:** Zi-Kun Yu, Xu-Yin Chen, Si-Han Liu, You-Ping Liu, Rui You, Pei-Yu Huang

**Affiliations:** ^1^ Department of Nasopharyngeal Carcinoma, Sun Yat-sen University Cancer Center, Guangzhou, China; ^2^ State Key Laboratory of Oncology in South China, Collaborative Innovation Center for Cancer Medicine, Guangdong Key Laboratory of Nasopharyngeal Carcinoma Diagnosis and Therapy, Sun Yat-sen University Cancer Center, Guangzhou, China

**Keywords:** nasopharyngeal carcinoma, anti-EGFR agents, concurrent chemotherapy, survival outcome, adverse events

## Abstract

**Objective:**

Anti-EGFR Targeted agents were found to be capable of modulating the antitumor immunity in head and neck cancer and become more and more frequently used in the treatment of nasopharyngeal carcinoma(NPC). We aimed to explore whether adding concurrent chemotherapy influences the survival outcome of patients with stage II-IVb NPC treated with concurrent anti-EGFR agents and intensity-modulated radiation therapy (IMRT) and explore other prognostic factors for the patients.

**Materials and Methods:**

A total of 656 stage II-IVb NPC patients treated with concurrent anti-EGFR agents plus IMRT between January 2011 and November 2015 were enrolled. Firstly, from these patients, a well-balanced cohort of 302 patients who received concurrent chemotherapy was created by matching potential prognostic factors. Furthermore, for all 656 stage II-IVb NPC patients, univariate and multivariate analyses of overall survival (OS), progression-free survival (PFS), locoregional recurrence-free survival (LRRFS) and distant metastasis-free survival (DMFS) were conducted to identify prognostic factors and to confirm the findings from the matching cohort.

**Results:**

Compared with concurrent anti-EGFR agents alone, combining concurrent cisplatin and anti-EGFR agents significantly improved the OS (5-year 94.7% versus 84.3%, P=0.012) and PFS (5-year 82.0% versus 71.7%, P=0.039) of NPC patients with more severe hematologic toxicity and mucositis. The independent prognostic factors identified by multivariate analysis of OS and PFS included concurrent chemotherapy, epstein-barr virus(EBV) status and clinical stage. Patients treated without induction chemotherapy (IC) may achieve more benefits from the addition of concurrent chemotherapy to concurrent anti-EGFR agents.

**Conclusions:**

For stage II-IVb NPC patients treated with concurrent anti-EGFR agents, the addition of concurrent chemotherapy can significantly improve the survival outcome.

## Introduction

Nasopharyngeal carcinoma (NPC) is an endemic disease in various regions, especially in southern China, where undifferentiated nasopharyngeal carcinoma occurs most frequently (1). In recent years, radiotherapy combined with chemotherapy has become the standard of care.

Although radiotherapy techniques, such as IMRT and a combination of chemotherapy, have been developed, distant metastasis and local or regional recurrence can still cause treatment failure. Approximately 20% of patients develop distant metastasis or locoregional recurrence (2, 3). In addition, researchers have shown that a combination of chemotherapy may cause an increased incidence of side effects, especially myelosuppression and gastrointestinal reactions (4, 5). The identification of a method to enhance the efficacy and alleviate the side effects of cancer treatment has recently become a research hotspot.

Epidermal growth factor receptor (EGFR), representing a promising new therapeutic target in cancer, is highly expressed in most human epithelial carcinomas and is expressed in 89%-100% of the samples collected from NPC patients (6, 7). Researchers also found that anti-EGFR Targeted agents can modulate the antitumor immunity in head and neck cancer (8), showing the close relationship between cancer immunity and anti-EGFR agents. Therefore, anti-EGFR targeted treatment, such as cetuximab (CTX) and nimotuzumab (NTZ), has become a potential treatment for NPC. Large retrospective studies have demonstrated that concurrent anti-EGFR therapy provides similar survival benefits with fewer toxicities, such as hematologic toxicities and gastrointestinal reactions, than concurrent chemotherapy for NPC patients (9). Furthermore, a series of studies have shown that combining anti-EGFR therapy and concurrent chemoradiotherapy can improve the prognosis of patients compared to concurrent chemoradiotherapy alone (10–12). Thus, in the Chinese Society of Clinical Oncology (CSCO) guidelines of NPC in 2020, concurrent anti-EGFR agents alone or a combination of concurrent anti-EGFR agents and chemotherapy were both recommended (category 3 recommendation) (13). Concurrent anti-EGFR therapy (Target-RT) is increasingly frequently used in the treatment of NPC.

Currently, concurrent cisplatin-based chemotherapy and anti-EGFR agents are both common therapies for stage II-IVb NPC patients treated with radiation therapy. However, no large cohort study focusing on the prognostic factors of stage II-IVb NPC patients treated with anti-EGFR agents and IMRT, especially studies investigating whether adding extra concurrent chemotherapy to concurrent anti-EGFR therapy can significantly improve the survival of patients or not.

Therefore, in the present study, we investigated the role of additional concurrent chemotherapy by comparing Target-RT and Target-RT plus concurrent cisplatin-based chemotherapy (CDDP) in terms of efficacy and safety directly so that we could explore whether extra concurrent chemotherapy is essential or not. In addition, we analyzed the prognostic factors of stage II-IVb nasopharyngeal carcinoma patients treated at the Sun Yat-sen University Cancer Center with concurrent anti-EGFR targeted therapy to confirm the findings from the matching cohort.

## Materials and Methods

### Patient Selection and Material Collection

Patients enrolled in this study were selected from newly diagnosed stage II-IVb NPC patients at Sun Yat-sen University Cancer Center. The stage of the patients was assessed according to the American Joint Committee on Cancer–Union for International Cancer Control 7th edition stage-classification system (14). The inclusion criteria included the following: (1). Diagnosed between January 2011 and November 2015. (2). Pathologically confirmed nonkeratinized differentiated or undifferentiated type of nonkeratinized carcinoma (WHO II or III). (3). Radiotherapy was given with at least one cycle of cetuximab (CTX) or nimotuzumab (NTX). (4). The radiation was delivered by IMRT.

The exclusion criteria were as follows: (1). Patients without complete clinical data, such as baseline body mass index (BMI), pretreatment plasma EBV DNA (EBV), C-reactive protein (CRP) and lactic dehydrogenase (LDH). (2). Patients who did not complete the planned dose of radiation therapy (RT). (3). Patients treated with adjuvant chemotherapy, noncisplatin-based concurrent chemotherapy or concurrent chemotherapy with more than one kind of drugs. (4) Patients diagnosed with a previous malignancy or other concomitant malignant disease. The patients were treated with IMRT according to the National Comprehensive Cancer Network (NCCN) guidelines, CSCO guidelines and treatment policies for NPC at Sun Yat-sen University Cancer Center (15, 16). The details of the treatment are included in the **Supplementary Appendix**.

Induction chemotherapy(IC) is playing an increasingly important role in the treatment of stage II-IVb nasopharyngeal carcinoma in the IMRT era, since it helps improve distant control and subsequent survival (12, 17). For the patients who received IC(375/656, 57.2%), patients received at least two cycles of IC, which included three different cisplatin combinations: taxane (159/375, 42.4%), fluorouracil (117/375, 31.2%), and taxane and fluorouracil (99/375, 26.4%). The details of chemotherapy are included in the **Supplementary Appendix**.

**Figure 1:**
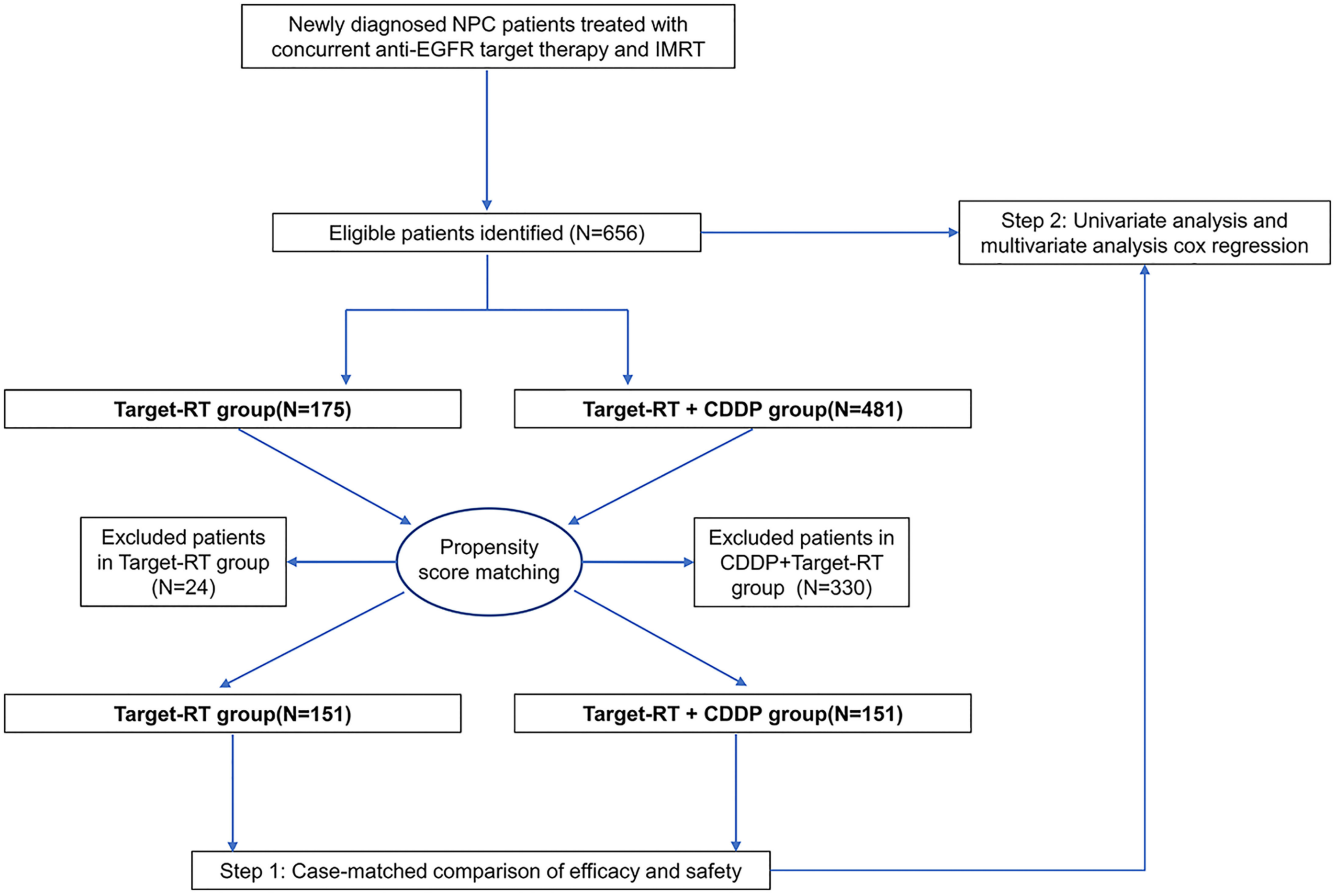
Patient selection and research flow chart.

All patient data were retrospectively collected before any treatment was initiated. The stage of patients was evaluated according to the seventh edition of the American Joint Committee on Cancer (AJCC) Staging System for nasopharyngeal carcinoma. Among the entire group of 656 patients, 481 patients received IMRT plus concurrent NTZ or CTX and CDDP, while 175 patients did not receive CDDP **(** **Figure 1** **)**. Using propensity scores to adjust for potentially significant factors, we created a well-balanced cohort by matching each patient treated with IMRT plus concurrent NTZ or CTX with a patient who received IMRT plus CDDP and concurrent NTZ or CTX. The variables available for propensity score matching included age (continuous variable), sex (male versus female), T stage (T3–4 versus T1–2), N stage (N2–3 versus N0–1), clinical stage (stage IV versus stage II-III), baseline pretreatment serum lactate dehydrogenase (LDH) level (continuous variable), pretreatment C-reactive protein (continuous variable), pretreatment body mass index (BMI) (continuous variable), pretreatment plasma EBV DNA copies (≥4000 versus <4000), IC status (patients receiving IC versus patients not receiving IC) and target-RT agents (NTX versus CTX) selected based on a previous study (18–22). The cutoff value of pretreatment plasma EBV was 4000 copies/mL according to routine clinical practice and a previous study (23). BMI was defined as pretreatment weight (kg) divided by the square of height (meters). We selected a total of 302 patients, including 151 patients in the Target-RT group and 151 patients in the Target-RT plus CDDP group (**Table S1**). Acute toxicities were defined as those occurring either within 90 days of the completion of IMRT or during the course of IMRT. Chemotherapy-related toxic effects and radiotherapy-related acute toxic effects were evaluated based on the Common Terminology Criteria for Adverse Events (version 4.0). Radiotherapy-related acute toxic effects were evaluated based on the Radiation Morbidity Scoring Criteria of the Radiation Therapy Oncology Group.

### Follow-Up and End-Points

After treatment, patients were monitored through phone and outpatient clinic follow-up visits. The eligible patients were followed up every 3 months for the first 3 years and then every 6 months until the fifth year. The intervals between follow-ups gradually increased to 12 months after 5 years. The location and timing of tumor recurrence and metastasis were documented. The primary endpoint of the study was overall survival (OS), and the secondary endpoints included progression-free survival (PFS), distant metastasis-free survival (DMFS) and locoregional recurrence-free survival (LRRFS). All intervals were calculated from the date of the end of radiation therapy. OS was defined as the time until death from any cause. PFS was defined as the time until the date of death from any cause or treatment failure caused by locoregional recurrence or distant metastasis of NPC, whichever occurred first. DMFS was defined as the time until the first distant metastasis. LRRFS was defined as the time until the first recurrence in the cervical and/or nasopharyngeal region after radiotherapy.

### Statistical Analysis

We first conducted a comparison between the IMRT plus CTX/NTZ and IMRT plus CTX/NTZ and concurrent cisplatin-based chemotherapy arms in terms of efficacy and safety in the well-balanced cohort of 302 patients. In addition, we conducted univariate and multivariate analyses based on all 656 cases. All analyses were performed using SPSS version 25.0 software, and a 2-tailed P value of less than 0.05 was considered statistically significant. The clinical characteristics of the cohort are described in **Table S1**, whereas the differences in these characteristics between the Target-RT plus CDDP arm and Target-RT arm were compared. Categorized variables were compared using the χ2 test, the correction χ2 test, or Fisher’s exact test, whereas continuous variables were compared using the Mann–Whitney U test. Survival outcome was calculated using the Kaplan–Meier method, and differences were compared by means of the log-rank test.

The potentially significant variables available for the univariate analysis included age, sex, T stage, N stage, clinical stage, baseline pretreatment serum LDH level, pretreatment CRP level, pretreatment BMI, pretreatment plasma EBV DNA copies, IC status, target-RT agents and CDDP status (the addition of CDDP or not). A multivariable Cox regression method was used to adjust for various covariates to identify independent prognostic factors for the survival outcome. The factors in the univariate analysis that were potentially significant (p<0.10) for OS, PFS, DMFS, or LRRFS were included in the multivariate Cox proportional hazards model of OS, PFS, DMFS, or LRRFS. Subgroup analysis was also performed to compare the efficacy of adding CDDP in different subgroups.

## Results

### Baseline Characteristics

The well balanced cohort of 302 patients who received anti-EGFR therapy with complete clinical data were included **(** **Figure 1** **)**. All of the patients received cetuximab (CTX) or nimotuzumab (NTX), and most patients (96.0%) received CTX or NTX every week for 5–8 cycles concurrent with radiation therapy. Both the Target-RT group and CDDP plus Target-RT group comprised 151 patients. The baseline characteristics of the patients are summarized in **Table S1** in the **Supplementary Appendix**. Age, total radiotherapy dose to the nasopharynx, baseline LDH, CRP, and BMI were continuous variables, whereas the other factors were categorized variables. No significant differences in these characteristics were found between the Target-RT plus CDDP arm and Target-RT arm.

In the Target-RT group, the median age of the patients was 50 years (range 18–74), and 80.1% of the patients were male. A total of 27.2% of patients suffered from stage IV NPC, and the pretreatment plasma EBV DNA copies of 67 (44.4%) patients were present at greater than 4000 copies/mL. NTX (nimotuzumab) was more frequently used (124/151, 82.1%) than CTZ (cetuximab) (27/151, 17.9%).

In the CDDP plus Target-RT group, patients receiving cisplatin at either 40 mg/m2 weekly or 100 mg/m2 every 3 weeks are acceptable (24–26), while 84.1%(127/151) of patients received 1-3 cycles of CDDP every 3 weeks. On the other hand, 15.9%(24/151) of the patients received 4-7 cycles of CDDP weekly. In total, 90.7%(137/151) of patients received at least 200 mg/m2 of CDDP. The median age of the patients was 49 years (range 10–72), and 81.5% of the patients were male. A total of 31.8% of the patients suffered from stage IV NPC, and the pretreatment plasma EBV DNA copies of 77/151 (51.0%) patients were present at greater than 4000 copies/mL. NTX was more frequently used (113/151, 74.8%) than CTZ (38/151, 25.2%).

### Survival Outcomes

The last follow-up visit was in October 2020 with a median follow-up period of 67.5 months (95% confidence interval (CI): 66.1–68.9 months). For the entire patient population, 38 deaths (27 in the Target-RT group and 11 in the CDDP plus Target-RT group) were reported. A total of 83 progressive cases were reported, of which 50 were in the Target-RT group and 33 were in the CDDP plus Target-RT group.

**Figure 2:**
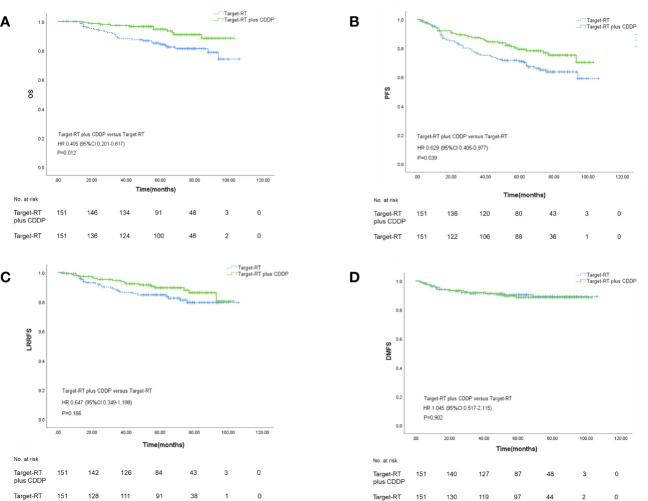
Kaplan–Meier curves of overall survival **(A)**, progression–free survival **(B)**, locoregional recurrence-free survival **(C)**, and distant metastasis-free survival **(D)** according to Target-RT plus CDDP or Target-RT treatment in the 302 well-balanced cohort.

The survival outcomes were described in **Figure 2**. Patients in the Target-RT plus CDDP group achieved better survival outcomes in both OS and PFS. The 5-year OS rates in the Target-RT plus CDDP group and Target-RT group were 94.7% and 84.3%, respectively (P=0.012). In regard to PFS, the 5-year PFS rates in the Target-RT plus CDDP group and Target-RT group were 82.0% and 71.7% (P=0.039), respectively, showing that CDDP can significantly improve the survival outcome even though the patients have been treated with concurrent anti-EGFR targeted therapy. The analysis of LRRFS and DMFS did not show the significant efficacy of CDDP in survival outcomes. The 5-year LRRFS rates in the Target-RT plus CDDP group and Target-RT group were 89.6% and 84.8%, respectively (P=0.166), whereas the 5-year DMFS rates in the Target-RT plus CDDP group and Target-RT group were 88.6% and 90.3%, respectively (P=0.902).

### Toxicity of Different Treatment Regimens

The χ2 test, corrected χ2 test or Fisher’s exact test was applied to analyze the differences in acute toxicities caused by the treatment. The acute toxicities in the NPC patients are summarized in **Table 1**. More patients in the concurrent chemotherapy subgroup suffered grade 3–4 hematologic toxicity (40.4% *versus* 19.8% P<0.001), especially leukopenia (31.8% versus 15.2%, P=0.001), although the P value of the χ2 test for thrombocytopenia, neutropenia and anemia was not significant. In addition, more grade 3-4 mucositis was found in the Target-RT plus CDDP group compared with the group treated without concurrent chemotherapy (28.5% versus 13.2% P=0.001).

**Table 1:** Acute toxicities in NPC patients receiving different treatment regimens.

	Target-RT without concurrent chemotherapy	Target-RT plus concurrent chemotherapy	P value
**Skin reaction**			ns
**G0-1**	136 (90.1%)	129 (85.4%)	
**G2**	14 (9.2%)	17 (11.3%)	
**G3**	1 (0.7%)	4 (2.6%)	
**G4**	0 (0%)	1 (0.7%)	
**Skin rash**			ns
**G0-1**	142 (94.0%)	138 (91.4%)	
**G2**	7 (4.6%)	10 (6.6%)	
**G3**	2 (1.4%)	3 (2.0%)	
**G4**	0 (0%)	0 (0%)	
**Thrombocytopenia**			ns
**G0-1**	138 (91.4%)	115 (76.2%)	
**G2**	11 (7.3%)	28 (18.5%)	
**G3**	2 (1.3%)	7 (4.6%)	
**G4**	0 (0%)	1 (0.7%)	
**Anemia**			ns
**G0-1**	135 (89.4%)	108 (71.5%)	
**G2**	15 (9.9%)	40 (26.5%)	
**G3**	1 (0.7%)	3 (2.0%)	
**G4**	0 (0%)	0 (0%)	
**Neutropenia**			ns
**G0-1**	106 (70.2%)	74 (49.0%)	
**G2**	16 (10.6%)	35 (23.2%)	
**G3**	9 (6.0%)	22 (14.6%)	
**G4**	20 (13.2%)	20 (13.2%)	
**Leukopenia**			0.001
**G0-1**	104 (68.9%)	50 (33.1%)	
**G2**	24 (15.9%)	53 (35.1%)	
**G3**	20 (13.2%)	42 (27.8%)	
**G4**	3 (2.0%)	6 (4.0%)	
**Hematological toxicity**			<0.001
**G0-1**	88 (58.3%)	37 (24.5%)	
**G2**	33 (21.9%)	53 (35.1%)	
**G3**	10 (6.6%)	40 (26.5%)	
**G4**	20 (13.2%)	21 (13.9%)	
**Mucositis**			0.001
**G0-1**	0 (0.0%)	0 (0.0%)	
**G2**	131 (86.8%)	108 (71.5%)	
**G3**	20 (13.2%)	40 (26.5%)	
**G4**	0 (0%)	3 (2.0%)	
**Nausea**			ns
**G0-1**	130 (86.1%)	88 (58.3%)	
**G2**	21 (13.9%)	59 (39.0%)	
**G3**	0 (0%)	3 (2.0%)	
**G4**	0 (0%)	1 (0.7%)	
**Vomiting**			ns
**G0-1**	137 (90.7%)	97 (64.2%)	
**G2**	14 (9.3%)	49 (32.5%)	
**G3**	0 (0%)	4 (2.6%)	
**G4**	0 (0%)	1 (0.7%)	
**Diarrhea**			ns
**G0-1**	150 (99.3%)	146 (96.7%)	
**G2**	0 (0%)	2 (1.3%)	
**G3**	1 (0.7%)	1 (0.7%)	
**G4**	0 (0%)	2 (1.3%)	
**Hepatoxicity**			ns
**G0-1**	142 (94.0%)	130 (86.1%)	
**G2**	7 (4.6%)	18 (11.9%)	
**G3**	2 (1.4%)	3 (2.0%)	
**G4**	0 (0%)	0 (0%)	
**Nephrotoxicity**			ns
**G0-1**	150 (99.3%)	149 (98.7%)	
**G2**	1 (0.7%)	2 (1.3%)	
**G3**	0 (0%)	0 (0%)	
**G4**	0 (0%)	0 (0%)	

Ns indicate that that the P value of the χ2 test, corrected χ2 test or Fisher’s exact test is not significant.

### Univariate and Multivariate Analysis of the Potential Prognostic Factors in the Entire Group

The results of univariate analysis are summarized in **Table S2** in the supplementary appendix. The potentially significant prognostic factors for OS included age, the addition of CDDP, AJCC clinical stage, T stage, N stage, pretreatment LDH, and pretreatment EBV status (≥4000 versus <4000). Age, the addition of CDDP, the addition of induction chemotherapy, AJCC clinical stage, N-stage, pretreatment LDH, and pretreatment EBV status were significant factors predicting the PFS benefits of the therapy. AJCC clinical stage, the addition of induction chemotherapy, and pretreatment EBV status were potential prognostic factors for LRRFS, whereas AJCC clinical stage, N-stage, LDH, and pretreatment EBV status were potential prognostic factors for DMFS.

Based on univariate analysis results, we performed multivariate analysis and the results are summarized in **Table 2**. Variables involved in the multivariate Cox regression analysis were selected according to the results shown in **Table S2**. Univariate analysis found that clinical stage plays a more significant role than T stage or N stage in the prognosis of patients. Due to the significant correlation between clinical stage and T/N stage, we used the AJCC clinical disease stage instead of the T/N stage in the Cox regression analysis. The potentially significant factors included age, the addition of concurrent chemotherapy, the addition of induction chemotherapy, AJCC clinical stage, LDH, and pretreatment EBV status (≥4000 versus <4000).

**Table 2:** Multivariate analysis for long-term survival for the entire group of NPC patients treated with concurrent anti-EGFR agents.

	OS		PFS		LRRFS		DMFS	
	HR (95%CI)	P	HR (95%CI)	P	HR (95%CI)	P	HR (95%CI)	P
Age	1.026 (1.004-1.048)	0.018	1.002 (0.988-1.016)	0.779	0.998 (0.977-1.018)	0.810	0.982 (0.962-1.002)	0.077
Target-RT plus CDDP versus Target-RT	0.359 (0.212-0.610)	<0.001	0.483 (0.339-0.689)	<0.001	0.551 (0.330-0.921)	0.023	0.718 (0.405-1.273)	0.257
Induction chemotherapy versus no induction chemotherapy	0.598 (0.347-1.030)	0.064	0.994 (0.693-1.427)	0.975	1.124 (0.662-1.907)	0.665	0.866 (0.513-1.463)	0.591
Stages IV versus stages II-III	6.270 (3.515-11.187)	<0.001	2.428 (1.711-3.444)	<0.001	2.505 (1.511-4.153)	<0.001	2.305 (1.388-3.827)	0.001
Pretreatment EBV DNA ≥4000 versus <4000	2.243 (1.309-3.844)	0.003	2.250 (1.589-3.186)	<0.001	1.709 (1.050-2.781)	0.031	2.510 (1.484-4.245)	0.001
LDH	1.003 (0.999-1.007)	0.136	1.001 (0.998-1.004)	0.417	0.996 (0.990-1.001)	0.117	1.003 (0.999-1.006)	0.117

IC, induction chemotherapy; HR, hazard ratio; 95% CI, 95% confidence interval.

According to the multivariate analysis, the treatment status of patients receiving concurrent chemotherapy was a significant prognostic factor for OS and PFS (HR 0.359, 95% CI 0.212-0.610, P<0.001 for OS and HR 0.483, 95% CI 0.339-0.689, P<0.001 for PFS). Adding concurrent chemotherapy was also found to be a favorable prognostic factor for LRRFS(HR 0.551, 95% CI 0.330-0.921, P=0.023). Moreover, pretreatment plasma EBV DNA copies and AJCC clinical stage were prognostic factors for PFS, OS, LRRFS, and DMFS (HR>1, P<0.05), predicting the poor survival outcome of patients with high pretreatment plasma EBV DNA copies and advanced clinical stage NPC.

### Subgroup Analysis of Adding Concurrent Chemotherapy and the Interaction Effect

**Figure 3:**
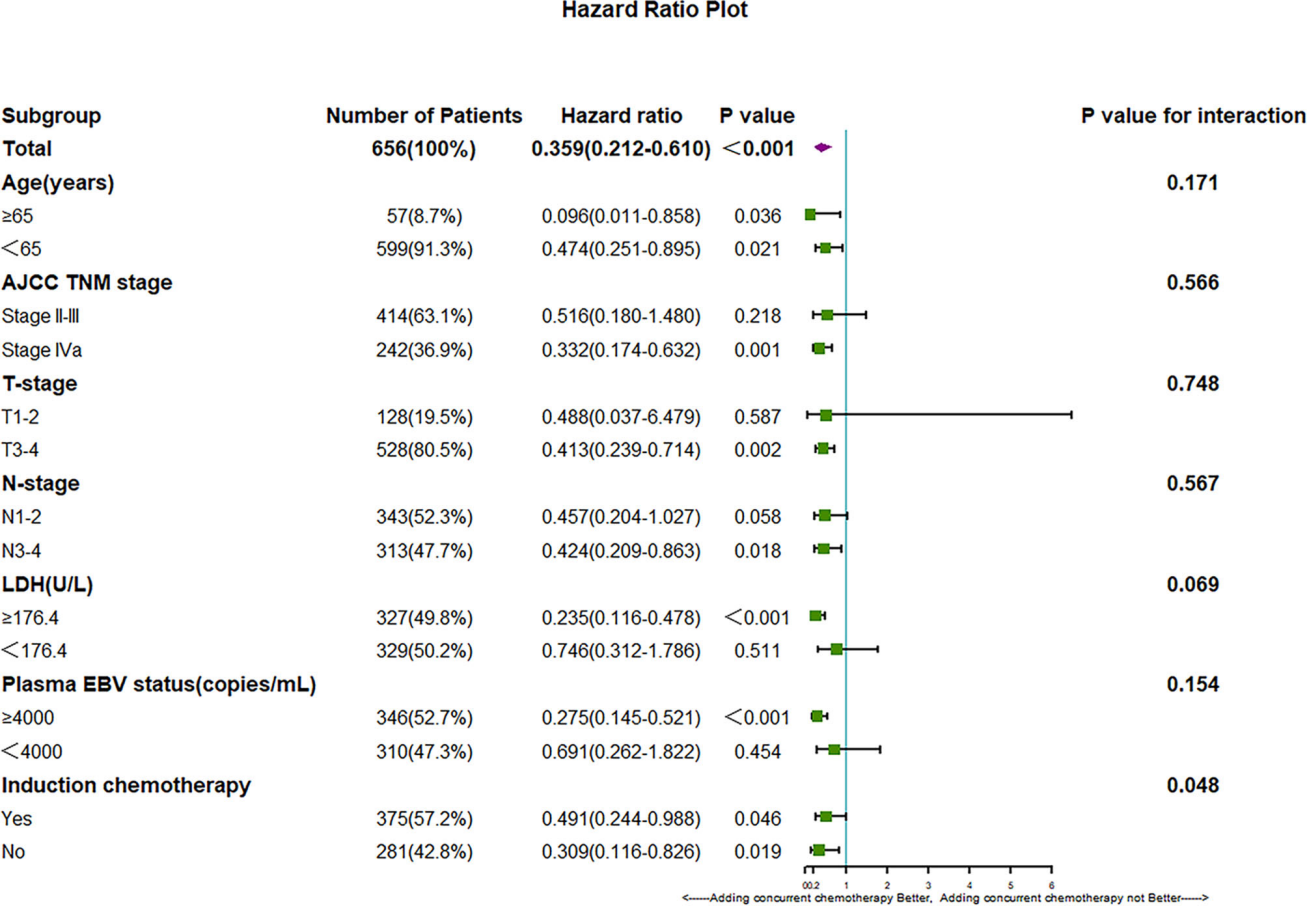
Hazard ratios for death and the interaction of the addition of CDDP with age, tumor stage, LDH, plasma EBV DNA copies and IC status.

Based on the results shown in **Table 2**, the subgroup analysis and the interaction between adding concurrent chemotherapy and other potentially significant prognostic factors for OS were studied to explore whether adding concurrent chemotherapy only benefits a specific subgroup of patients. The cutoff value of pretreatment plasma EBV was 4000 copies/mL according to routine clinical practice and a previous study (23). The cutoff value of pretreatment LDH was the median of pretreatment plasma LDH in the entire group of 656 patients, which is 175.4U/L. The results of the subgroup analysis are summarized in **Figure 3**.

We found that subgroup analysis of OS showed a significant interaction between induction chemotherapy and the efficacy of CDDP (P for interaction: 0.048). These results demonstrate that for patients without induction chemotherapy, the addition of CDDP can provide more survival benefits; however, the addition of CDDP can improve OS outcome in both subgroups regardless of induction chemotherapy (P=0.019 in the subgroup without IC and P=0.046 in the subgroup with IC). In addition, we found that different pretreatment LDH levels may affect the efficacy of extra CDDP. This finding indicates that patients with higher pretreatment LDH level may gain more benefits by adding concurrent chemotherapy; however, the P value of the interaction was not significant (P value for interaction between LDH and extra CDDP 0.069).

## Discussion

Concurrent cisplatin-based chemotherapy and anti-EGFR agents are both common therapies for stage II-IVb NPC patients treated with radiation therapy. Large retrospective studies have demonstrated that for NPC patients, anti-EGFR therapy has similar benefits as chemotherapy but with less severe toxicity, such as hematologic toxicities and gastrointestinal reactions (9). In addition, a series of retrospective studies have shown that adding anti-EGFR therapy to concurrent chemotherapy can enhance the efficacy of treatment for stage II-IVb NPC patients (10–12, 27, 28). However, the efficacy of adding chemotherapy to concurrent anti-EGFR therapy has not been well studied. Our research in the well-balanced cohort found that when compared to concurrent anti-EGFR therapy alone, adding concurrent chemotherapy significantly improved the PFS and OS of stage II-IVb NPC patients (P=0.039 for PFS and P=0.012 for OS). In addition, multivariate analysis of the entire group confirmed the efficacy of concurrent chemotherapy, which was also noted in the well-balanced cohort (P<0.001 for both PFS and OS). Adding concurrent chemotherapy was also a favorable prognostic factor for LRRFS (HR 0.551, 95% CI 0.330-0.921, P=0.023), showing that the addition of concurrent chemotherapy may improve PFS by improving LRRFS. Consistent with previous research (23), pretreatment plasma EBV DNA copies were an independent prognostic factor for OS (P=0.003), PFS (P<0.001), LRRFS (P=0.031) and DMFS (P=0.001), demonstrating the feasibility of the cutoff value of 4000 copies/ml to distinguish high-risk and low-risk patients independent of their clinical stage.

In the subgroup analysis of OS, induction chemotherapy was found to significantly influence the efficacy of adding concurrent chemotherapy (P value for the interaction in OS 0.048). These results demonstrate that patients treated without induction chemotherapy may achieve more survival benefits by adding concurrent chemotherapy. However, we also found that regardless of whether they receive induction chemotherapy, combining CDDP with Target-RT can significantly improve the survival outcome of NPC patients (P=0.019 for OS in the Target-RT group, respectively; P=0.046 for OS in the IC plus Target-RT group, respectively). These results demonstrate the powerful efficacy and the necessity of adding extraconcurrent chemotherapy to anti-EGFR therapy. In addition, patients with a high level of high pretreatment LDH may gain more benefits from adding concurrent chemotherapy; however, the P value for interaction was not significant (P value for interaction of adding concurrent chemotherapy and LDH status in OS: 0.069). These results demonstrate the potential predictive value of pretreatment LDH for the selection of suitable patients to receive concurrent chemotherapy, and this topic is worthy of further study. In addition, the subgroup analysis of age shows that even for elderly patients over 65 years old (P=0.036 in OS), adding concurrent chemotherapy can achieve better survival outcomes, which indicates that age is not a suitable criterion for the selection of appropriate patients adding extra concurrent chemotherapy to Target-RT. In conclusion, the subgroup analysis of OS suggested that the benefit of CDDP was not limited to a particular subgroup of patients and confirmed the necessity of adding CDDP to Target-RT. Previous study have demonstrated that concurrent chemotherapy may benefit elder patients (29, 30), while some researchers argued that elder patients may not gain survival benefits by adding concurrent chemotherapy (31). Although we found that adding CDDP to Target- RT can improve survival outcomes, the application of CDDP for elder patients should be considered carefully, because we excluded patients who did not complete the planned dose of radiation therapy, while the treatment interruptions may be caused by the severe side effects of additional CDDP.

When it comes to the side effects of additional CDDP, our research also evaluated the toxicity of different treatment regimens. Consistent with previous research (4, 5), we observed that the addition of concurrent chemotherapy led to more grade 3-4 hematologic toxicity (40.4% versus 19.8% P<0.001), leukopenia (31.8% versus 15.2%, P=0.001), and mucositis (28.5% versus 13.2% P=0.001). Although the addition of concurrent chemotherapy can significantly improve survival outcomes, the severe side effects caused by concurrent chemotherapy are still an insurmountable problem.

Based on the serious side effects of concurrent chemotherapy and the fewer side effects caused by anti-EGFR target therapy, identifying patients who are suitable for omitting concurrent chemotherapy has become an emerging question that is worthy of exploration. Based on the subgroup analysis of OS, we found that pretreatment LDH status may influence the survival benefit of additional CDDP(P for interaction: 0.069). However, we also found that both patients with low or high LDH level may receive better OS outcome in Target-RT plus CDDP group when compared to Target-RT group(HR=0.235 for high LDH level group, while HR=0.746 for low LDH level group), although the P-value in the low LDH level group was 0.511. How to identify patients who are suitable for omitting concurrent chemotherapy should be studied further in larger cohort of study.

One limitation of our study is that our study was a single-center retrospective study in a high-NPC prevalence area. Additionally, although we eliminated some biases by multivariate analysis with an adjusted Cox proportional hazards model and propensity score matching, the presence of other confounding factors in our study was unclear. Additional multicenter prospective investigations are warranted to evaluate the efficacy and safety of adding concurrent chemotherapy to concurrent anti-EGFR agents for NPC patients.

## Conclusion

In summary, the addition of concurrent chemotherapy can significantly improve the survival outcome of stage II-IVb nasopharyngeal carcinoma patients treated with concurrent anti-EGFR agents.

## Data Availability Statement

The raw data supporting the conclusions of this article will be made available by the authors, without undue reservation.

## Ethics Statement

The studies involving human participants were reviewed and approved by Clinical Research Committee of Sun Yat-Sen University Cancer Center. Written informed consent to participate in this study was provided by the participants or the participants’ legal guardian/next of kin if the participant was under legal age of adults.

## Author Contributions

ZK-Y and RY participated in study design. ZK-Y, XY-C, and SH-L were responsible for the analysis of data. ZK-Y and YP-L were responsible for drafting the manuscript. PY-H put forward the concepts of the study. All the authors revised the manuscript. All the authors approved the final manuscript.

## Funding

This work was supported by the Program of Sun Yat-Sen University for Clinical Research 5010 Program (No.2018015), the National Natural Science Foundation of China (No.81874134).

## Conflict of Interest

The authors declare that the research was conducted in the absence of any commercial or financial relationships that could be construed as a potential conflict of interest.

The reviewers CW and QZ have declared a shared parent affiliation with the authors at the time of review.

## Publisher’s Note

All claims expressed in this article are solely those of the authors and do not necessarily represent those of their affiliated organizations, or those of the publisher, the editors and the reviewers. Any product that may be evaluated in this article, or claim that may be made by its manufacturer, is not guaranteed or endorsed by the publisher.
