# Supplementary material for: Adding Concurrent Chemotherapy Significantly Improves the Survival of Stage II-IVb Nasopharyngeal Carcinoma Patients Treated With Concurrent Anti-EGFR Agents
Source: Front Oncol. 2021 Dec 17;11:814881. doi: 10.3389/fonc.2021.814881 (PMC8718697; doi:10.3389/fonc.2021.814881)
Supplement: Supplementary file 1 [file DataSheet_1.docx]

**Supplementary Appendix**

**Treatment details**

The induction chemotherapy (IC) was cisplatin-based chemotherapy. Combination of cisplatin with taxane, fluorouracil or taxane plus fluorouracil were given to patients, which was administered every 3 weeks for at least 2 cycles. For concurrent chemotherapy, cisplatin was given weekly or every 3 weeks on weeks 1, 4, and 7 in combination with intensity-modulated radiation therapy(IMRT). Patients receiving cisplatin at either 40 mg/m^2^ weekly or 100 mg/m^2^ every 3 weeks are acceptable([1-3](#_ENREF_1)). Concurrent nimotuzumab(NTX) was administered with IMRT at a dose of 200 mg weekly, which was diluted in 250 mL saline and intravenously infused over 1 hour. Cetuximab (CTX) was administered at an initial dose of 400 mg/m^2^ followed by 250 mg/m^2^ weekly concomitantly with IMRT.

All patients received IMRT([4](#_ENREF_4), [5](#_ENREF_5)). Target volumes were defined in accordance with the International Commission on Radiation Units and Measurements (ICRU) reports 50 and 62. Gross tumor volume (GTV) was defined as the gross tumor determined by physical examination, endoscopic findings and imaging (including MRI and PET/CT, if available). GTV included GTVnx and GTVnd. GTVnx included the sum of the primary tumor volume and the enlarged retropharyngeal nodes, while GTVnd was the volume of the involved gross cervical lymph nodes. The first clinical tumor volume (CTV1) which is the high-risk clinical target volume, was defined as the GTVnx plus a 5–10-mm margin (2–3 mm posteriorly) to encompass the high-risk sites of microscopic extension and the whole nasopharynx. The clinical target volume 2 (CTV2), which is the low-risk clinical target volume, was defined as the CTV1 plus a 5–10-mm margin (2–3 mm posteriorly) to encompass the low-risk sites of microscopic extension, the cervical level where the involved lymph nodes were located, the elective neck area from level II to Vb, and the supraclavicular fossae.

The prescribed doses were 66–70 Gy, 64–70 Gy, 60–62 Gy, and 54–56 Gy in 30–33 fractions for the PTVs derived from GTVnx, GTVnd, CTV1, and CTV2, respectively.

All patients were treated once daily with five fractions every week. Dose constraints to the critical structures were within the tolerance according to the RTOG 0225 protocol, and we tried our best to meet the criteria as closely as possible.

**References**

1. Szturz P, Wouters K, Kiyota N, Tahara M, Prabhash K, Noronha V, et al. Weekly Low-Dose Versus Three-Weekly High-Dose Cisplatin for Concurrent Chemoradiation in Locoregionally Advanced Non-Nasopharyngeal Head and Neck Cancer: A Systematic Review and Meta-Analysis of Aggregate Data. *Oncologist* (2017) 22(9):1056-66. Epub 2017/05/24. doi: 10.1634/theoncologist.2017-0015. PubMed PMID: 28533474; PubMed Central PMCID: PMCPMC5599190.

2. Zhu Q, Hu H, Tang LQ, You R, Zhao JJ, Weng DS, et al. Weekly versus triweekly cisplatin plus intensity-modulated radiotherapy in locally advanced nasopharyngeal carcinoma: A propensity score analysis with a large cohort. *J Cancer* (2018) 9(19):3447-55. Epub 2018/10/13. doi: 10.7150/jca.26110. PubMed PMID: 30310501; PubMed Central PMCID: PMCPMC6171032.

3. Wang K, Dong J, He S, Wang X, Jiang C, Hu P, et al. Comparison of weekly and triweekly cisplatin regimens during concurrent chemoradiotherapy for nasopharyngeal carcinoma. *BMC Cancer* (2019) 19(1):482. Epub 2019/05/24. doi: 10.1186/s12885-019-5688-z. PubMed PMID: 31117967; PubMed Central PMCID: PMCPMC6532163.

4. Lai SZ, Li WF, Chen L, Luo W, Chen YY, Liu LZ, et al. How does intensity-modulated radiotherapy versus conventional two-dimensional radiotherapy influence the treatment results in nasopharyngeal carcinoma patients? *Int J Radiat Oncol Biol Phys* (2011) 80(3):661-8. Epub 2010/07/21. doi: 10.1016/j.ijrobp.2010.03.024. PubMed PMID: 20643517.

5. Xiao WW, Huang SM, Han F, Wu SX, Lu LX, Lin CG, et al. Local control, survival, and late toxicities of locally advanced nasopharyngeal carcinoma treated by simultaneous modulated accelerated radiotherapy combined with cisplatin concurrent chemotherapy: long-term results of a phase 2 study. *Cancer* (2011) 117(9):1874-83. Epub 2011/04/22. doi: 10.1002/cncr.25754. PubMed PMID: 21509764.

**Table S1. Baseline characteristics in the 302 well-balanced cohort.**

| Baseline characteristics | Target-RT group  (n =151) | CDDP plus Target-RT group (n =151) | P value |
| --- | --- | --- | --- |
| Age |  |  | 0.064 |
| Median | 50.00 | 49.00 |  |
| Range | 18.00-74.00 | 10.00-72.00 |  |
| Gender(%) |  |  | 0.770 |
| Male | 121(80.1%) | 123(81.5%) |  |
| Female | 30(19.9%) | 28(18.5%) |  |
| Stages IV versus stages II-III(%) |  |  | 0.377 |
| II-III | 110(72.8%) | 103(68.2%) |  |
| IV | 41(27.2%) | 48(31.8%) |  |
| AJCC T-stage(%) |  |  | 0.900 |
| T1-2 | 44(29.1%) | 45(29.8%) |  |
| T3-4 | 107(70.9%) | 106(70.2%) |  |
| AJCC N-stage(%) |  |  | 0.906 |
| N0-1 | 92(60.9%) | 93(61.6%) |  |
| N2-3 | 59(39.1%) | 58(38.4%) |  |
| Adding induction chemotherapy or not |  |  | 0.729 |
| Induction chemotherapy plus target therapy | 81(53.6%) | 84(55.6%) |  |
| Target therapy alone | 70(46.4%) | 67(44.4%) |  |
| CRP(U/L) |  |  | 0.962 |
| Median | 1.97 | 1.80 |  |
| Range | 0.20-43.87 | 0.15-118.90 |  |
| LDH(mg/L) |  |  | 0.344 |
| Median | 173.30 | 178.70 |  |
| Range | 103.80-366.60 | 112.00- 507.60 |  |
| BMI(kg/m2) |  |  | 0.070 |
| Median | 23.24 | 24.19 |  |
| Range | 15.94-34.63 | 15.89-37.18 |  |
| Pretreatment EBV DNA copies(≥4000copies/ml versus＜4000 copies/ml) |  |  | 0.249 |
| ＜4000 copies/ml | 84(55.6%) | 74(49.0%) |  |
| ≥4000 copies/ml | 67(44.4%) | 77(51.0%) |  |
| Anti-EGFR agents |  |  | 0.124 |
| NTX (nimotuzumab) | 124(82.1%) | 113(74.8%) |  |
| CTZ (cetuximab) | 27(17.9%) | 38(25.2%) |  |

Target-RT: Concurrent anti-EGFR therapy; IC: induction chemotherapy; HR: hazard ratio; CDDP: concurrent cisplatin-based chemotherapy; NTZ: Nimotuzumab; CTX: Cetuximab.

**Table S2. Univariate analysis of other prognostic factors for the entire group of NPC patients treated with concurrent anti-EGFR agents.**

|  | OS | |  | PFS | |  | LRRFS | |  | DMFS | |
| --- | --- | --- | --- | --- | --- | --- | --- | --- | --- | --- | --- |
|  | HR(95%CI) | P |  | HR(95%CI) | P |  | HR(95%CI) | P |  | HR(95%CI) | P |
| Age | 1.045(1.023-1.067) | ＜0.001 |  | 1.012(0.998-1.026) | 0.098 |  | 1.002(0.982-1.022) | 0.845 |  | 0.985(0.965-1.005) | 0.142 |
| Gender  Male versus Female | 0.974(0.532-1.783) | 0.931 |  | 1.192(0.782-1.818) | 0.415 |  | 1.363(0.718-2.589) | 0.344 |  | 1.312(0.690-2.495) | 0.408 |
| Target-RT plus CDDP versus Target-RT | 0.437(0.271-0.704) | 0.001 |  | 0.611(0.441-0.848) | 0.003 |  | 0.699(0.431-1.131) | 0.145 |  | 1.065(0.624-1.818) | 0.817 |
| Induction chemotherapy versus no induction chemotherapy | 1.161(0.716-1.884) | 0.544 |  | 1.446(1.041-2.009) | 0.028 |  | 1.592(0.982-2.580) | 0.059 |  | 1.447(0.889-2.356) | 0.137 |
| Stages IV versus stages II-III | 4.765(2.844-7.984) | ＜0.001 |  | 2.505(1.822-3.444) | ＜0.001 |  | 2.537(1.602-4.019) | ＜0.001 |  | 2.504(1.565-4.005) | ＜0.001 |
| NTX versus CTX | 1.058(0.604-1.854) | 0.844 |  | 0.876(0.611-1.256) | 0.470 |  | 0.984(0.578-1.674) | 0.951 |  | 0.822(0.486-1.390) | 0.465 |
| T-stage  (T3-4 versus T1-2) | 3.394(1.364-8.442) | 0.009 |  | 1.143(0.758-1.723) | 0.523 |  | 0.863(0.501-1.486) | 0.595 |  | 1.231(0.662-2.291) | 0.511 |
| N-stage  (N2-3 versus N0-1) | 1.771(1.092-2.872) | 0.021 |  | 1.733(1.258-2.388) | 0.001 |  | 1.208(0.764-1.911) | 0.420 |  | 2.519(1.532-4.143) | ＜0.001 |
| CRP | 1.006(0.988-1.024) | 0.529 |  | 1.005(0.992-1.018) | 0.478 |  | 1.002(0.982-1.023) | 0.818 |  | 1.010(0.994-1.026) | 0.228 |
| BMI | 0.972 (0.904-1.045) | 0.447 |  | 1.020(0.974-1.069) | 0.404 |  | 1.045(0.979-1.116) | 0.184 |  | 1.002(0.935-1.074) | 0.960 |
| Pretreatment EBV DNA  ≥4000 versus＜4000 | 2.879(1.719-4.822) | ＜0.001 |  | 2.585(1.850-3.612) | ＜0.001 |  | 1.841(1.156-2.932) | 0.010 |  | 2.959(1.777-4.927) | ＜0.001 |
| LDH | 1.003(1.000-1.006) | 0.026 |  | 1.002(1.000-1.004) | 0.077 |  | 0.998(0.993-1.003) | 0.348 |  | 1.004(1.001-1.006) | 0.018 |

Target-RT: Concurrent anti-EGFR therapy; IC: induction chemotherapy; HR: hazard ratio; 95% CI: 95% confidence interval; CDDP: concurrent cisplatin-based chemotherapy.
